# Real-world Data on Risk Factors for Emergency Department Visits to Treat Outpatient Chemotherapy-Associated Toxicities

**DOI:** 10.1158/2767-9764.CRC-24-0631

**Published:** 2025-06-16

**Authors:** Sanja Karovic, Erik Dvergsten, Chiara Pierattini, Ana Barac, Lauren Fay, Timothy L. Cannon, Kathleen K. Harnden, Jeanny B. Aragon-Ching, Raymund S. Cuevo, Michael L. Maitland, John F. Deeken

**Affiliations:** 1 Inova Schar Cancer Institute, Fairfax, Virginia.; 2 Inova Schar Heart and Vascular, Inova Health System, Falls Church, Virginia.

## Abstract

The Centers for Medicare & Medicaid Services Hospital Outpatient Quality Reporting Program’s OP-35 rule penalizes health systems that have a higher-than-expected rate of emergency department (ED) visits or inpatient admissions for 10 potentially preventable conditions within 30 days of receiving chemotherapy. Identifying patients at risk for toxicities and resultant acute care could lead to reducing the rate of such events, improving patient care, and reducing costs. We identified patients with cancer seen in the ED at our institution between January 1, 2018, and December 31, 2021, for one of the OP-35 toxicities who had received chemotherapy within the previous 30 days and analyzed demographic factors using zero-truncated Poisson regression. We further analyzed comorbid conditions for risk factors by matching by demographics and cancer type a cohort of patients without ED visits due to OP-35 events. A total of 1,618 patients were identified. The most frequent events were pain, sepsis, and fever. Thirty-nine percent had two or more visits during the study, and among those patients, the most frequent cancer types were gastrointestinal (32%) and breast (22%) cancers. Race, age, and sex were associated with an increased risk of events. In the matched cohort analysis, five comorbidities were statistically significant (*P* < 0.05) with event risk: history of coagulopathy/pulmonary emboli, myocardial infarction, cardiac arrhythmias, depression, and weight loss (concordance = 0.58). Forty-seven percent of patients with an event had at least one of these five comorbidities. Future interventions may concentrate on resources to monitor patients based on the risk assessment established in this study.

**Significance:**

Cardiovascular comorbidities, cancer cachexia, and depression were associated with increased risk for ED visits due to OP-35 events throughout cancer treatment. Future interventions may concentrate on resources to monitor patients based on the risk assessment established in this real-world data study.

## Introduction

The treatment of solid tumor malignancies has moved dramatically from the inpatient to the outpatient ambulatory environment over the past several decades. This is in part due to the advances in supportive medications to treat the significant side effects that can be caused by cytotoxic chemotherapy. Common acute toxicities associated with almost all chemotherapeutic agents include myelosuppression and gastrointestinal (GI) toxicities such as nausea, vomiting, and diarrhea. If the toxicities become too severe, an evaluation in the emergency department (ED) or an inpatient admission might be necessary. This, in turn, can cause a significant decrease in quality of life and related burdens on patients with cancer and their families, as well as incur significant medical costs to the health system. As Shoemaker and colleagues (1) emphasized, effective symptom management extends beyond direct treatment, significantly enhancing patient quality of life and potentially reducing these admissions.

In one meta-analysis of 16 studies, the median rate of hospital admission of oncology patients from the ED was 58%, with rates ranging from 31% to 100% (2). In a study completed for the Centers for Medicare & Medicaid Services (CMS), oncology patients had the highest 30-day readmission rate compared with other patient groups at 25% (3). Many costly hospitalizations are unplanned, and upwards of 50% of these admissions may be preventable (4). Preventable admissions often result from issues such as inadequate outpatient monitoring, delayed intervention for emerging complications, and lack of patient education on managing symptoms at home.

Oncology care is a large and growing expenditure for the US healthcare system. The total cost is expected to grow from $183 billion in 2015 to $246 billion by 2030 (5). This increase is driven by several factors, including the increasing incidence of cancer due to an aging population, the high cost of new and advanced treatments, and the extended survival rates of patients with cancer, which necessitate prolonged and ongoing care. Acute inpatient hospital care is one of the highest contributors to variation in cancer care costs across the United States (6, 7). This variation can be attributed to differences in hospital practices, regional healthcare policies, and the availability of outpatient care resources. Hospitals incur significant expenses when managing acute toxicities that require inpatient care, further emphasizing the need for effective outpatient management strategies to control costs and improve patient outcomes.

The CMS Hospital Outpatient Quality Reporting Program instituted a final rule OP-35 to reduce preventable acute care needs for patients with cancer receiving outpatient chemotherapy, thereby saving resources (8). OP-35 aims to assess the quality of care for patients receiving chemotherapy and encourage performance improvement by assessing ED visits and inpatient admissions by patients with cancer for 10 potentially preventable conditions within 30 days of receiving chemotherapy (3). The 10 conditions are anemia, dehydration, diarrhea, emesis, fever, nausea, neutropenia, pain, pneumonia, or sepsis. The Hospital Quality Reporting Program is a pay-for-reporting program that mandates that the specified data be collected and submitted, or the participating hospital could face financial penalties through reduced Medicare payments. After data collection, CMS publishes emergency and hospital admission rates for patients with cancer at all participating hospitals (8). In 2021, the observed national rate of OP-35 admissions per 100 patients with cancer per year was 10.2.

Inova Health System is a nonprofit community health system in Northern Virginia with more than 24,000 employees and 5 hospitals serving the Washington D.C. metropolitan area. We have a large cancer program with about 8,800 new analytic cancer cases per year. We have a diverse population of patients with cancer, including by gender (62% female and 38% male), as well as race and ethnicity (58.1% White/Caucasian, 14.4% Black/African American, 11.7% Asian, and 3.4% Hispanic/Latino).

Our study aims to use real-world data from Inova Health System to identify demographic and comorbidity profiles that increase the likelihood of ED visits for OP-35 conditions among chemotherapy patients. By understanding these risk factors, we can develop and utilize targeted strategies to mitigate the need for acute care, thereby improving patient outcomes and reducing healthcare costs. We performed a retrospective cohort analysis of patients treated at Inova Schar Cancer Institute (ISCI), including the subset of patients who visited the ED for one or more of the 10 OP-35 conditions. Our investigation included assessing demographic factors and comorbidities to develop a comprehensive risk profile for patients likely to require acute care following outpatient chemotherapy treatment. This analysis will contribute to the ongoing efforts to enhance quality of care and reduce unnecessary hospitalizations in the oncology patient population.

## Materials and Methods

### Data collection

This institutional review board–approved study was a noninterventional, retrospective analysis of real-world data extracted from electronic health records of adult patients with cancer treated at a community hospital cancer program. The study period was from January 1, 2018, to December 31, 2021. Patients were included based on the following criteria: 18 years of age or older, receiving cancer treatment at ISCI, and having one of the 10 OP-35 diagnoses (anemia, dehydration, diarrhea, emesis, fever, nausea, neutropenia, pain, pneumonia, or sepsis) as the primary or secondary diagnosis at the time of an acute care evaluation within a 30-day period after receiving cancer treatment. For simplicity, an ED visit meeting these criteria will be referred to as an event. Patients with acute leukemia were excluded based on their high-toxicity treatment and recurrence of disease which leads to high ED events.

**Figure 1:**
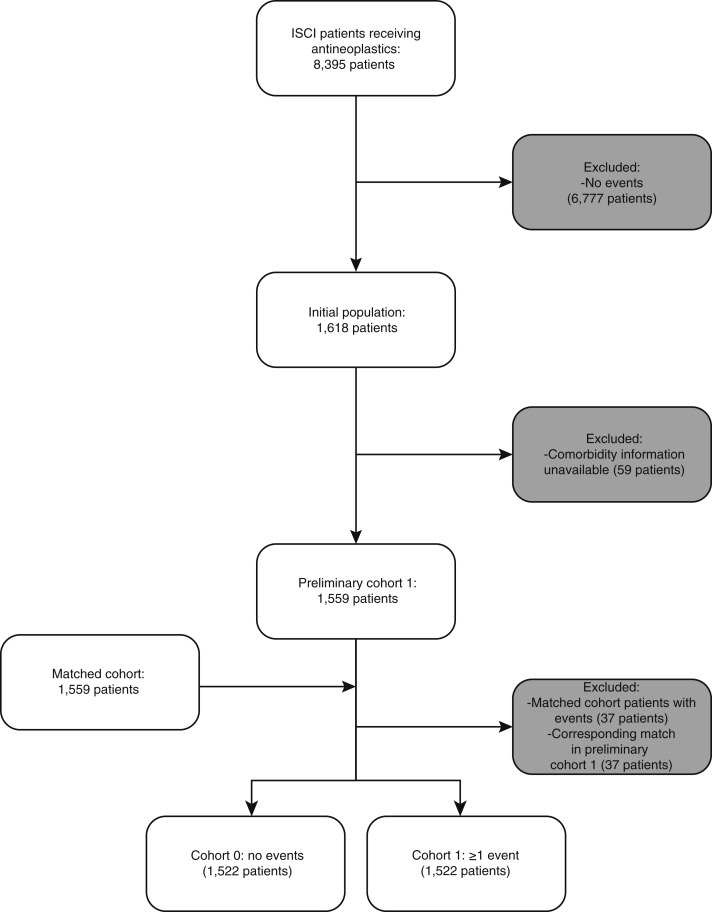
Patient selection flowchart.

For this initial population, we collected data on demographics, disease/comorbidities, and status (alive/deceased) at last follow-up. Patients with available comorbidity information and one or more events were labeled as cohort 1. Cohort 0, a comparison group with available comorbidity information but without events, was included for additional analyses. In a similar manner, we further analyzed a subset of cohort 1 and cohort 0 patients with highly clinically preventable events (defined as events due to pain, emesis, nausea, dehydration, or diarrhea). The patient selection flowchart is shown in Fig. 1.

### Data analysis

First, we analyzed event counts by demographic variables for the initial population. A zero-truncated Poisson regression analysis was performed in R v4.1.1 (RRID: SCR_001905; ref. 9). Variables included in the model were sex, age, and race. Age was determined by the age at a patient’s first cancer treatment during the study period. Race was self-reported. An event with multiple diagnoses was counted as one event.

Second, we performed risk analysis within comorbid conditions. In order to investigate whether medical comorbidities were associated with event risk, we matched this cohort (cohort 1) one to one to a second cohort (cohort 0) of patients with cancer by sex, age at first treatment, race, and cancer type who did not have events. Propensity score matching was performed using the R package MatchIt v4.5.1 (RRID: SCR_025618; ref. 10).

Comorbidities were converted from ICD-10 codes with the comorbidity R package v1.0.5 (11) using a combination of the categories present in the Charlson (12) and Elixhauser (13) comorbidity indices for a total of 32 comorbidities as potential important covariates. Conditional logistic regression with bidirectional stepwise variable selection was performed on the 11 important comorbidities. Additionally, two other machine learning models, random forest (RF) and support vector machines (SVM), were considered to classify a patient as having an event or not having an event. Training and test sets were split 70%/30%, respectively.

### Data availability

The data analyzed in this study are not publicly available because of patient privacy requirements. Data are available upon reasonable request to the corresponding author.

## Results

### Initial population analysis

Data extraction revealed 8,395 patients who received antineoplastic therapy from an ISCI physician from January 1, 2018, through December 31, 2021. Patients who did not have an event during this time (*n* = 6,777) were excluded producing an initial population of 1,618 patients who had one or more ED visits for any of the 10 CMS OP-35 conditions within 30 days of chemotherapy (Fig. 1). The most frequent reasons for events were pain, sepsis, and fever. A total of 56% of patients were female with a median age of 64 years (range = 20–95). Patients who died during the study period were more likely to have had more events than patients who were still alive at the end of the study period.

There was a median of 40 days between the start of a new treatment regimen and an event. The timelines of when patients were sent to the ED compared with the start of a new regimen, by quartile, were as follows: 25% within 14 days, 50% within 40 days, and 75% within 93 days.

In terms of insurance coverage, 39% of patients had Medicare, 45% had commercial insurance, 8% were on Medicaid, and 7% received charity care. More than a third (39%) of patients had two or more visits during the 4-year span of the study, and among those patients, the most frequent cancer types were GI (32%) and breast (22%) cancers.

In a zero-truncated Poisson regression, a typical male in this study (62 years old, White/Caucasian, mean = 1.85 years of follow-up) was predicted to have 12% more visits than the typical female. Adjusting for the mean age at first treatment (62 years), male sex, and mean follow-up time of 1.85 years, Hispanic/Latino [2.06, 95% confidence interval (CI) 1.81, 2.40] and Black/African American (2.06, 95% CI 1.89, 2.27) patients were predicted to have more events than White/Caucasian (1.72, 95% CI 1.65, 1.80).

### Matched analysis

Patients without available comorbidity information were removed from the initial population to obtain cohort 1 and its corresponding matched group with no events, cohort 0 (*n* = 1,522 per cohort). Table 1 displays the baseline demographics of the full study population of the matched analysis (*n* = 3,044). The mean age at first cancer treatment was 62 years with 56% being female. The population was 58% White or Caucasian, whereas the next largest race was Asian at 16%. GI and breast cancers accounted for more than half of the population at 26% and 25%, respectively. Approximately 47% of patients had commercial insurance, whereas 38% had Medicare. GI physicians accounted for the most ED visits (Supplementary Table S1). A further subdivision of GI and genitourinary cancer is included in Supplementary Table S2.

**Table 1:** Baseline patient characteristics and demographics by occurrence of ED visits

Characteristic	Study population (*N* = 3,044), *n* (%)	Cohort 0: patients without events (*n* = 1,522), *n* (%)	Cohort 1: patients with events (*n* = 1,522), *n* (%)
Age at first treatment, years			
Mean (SD)	61.8 (14.4)	61.9 (14.7)	61.7 (14.1)
Sex			
Female	1,708 (56%)	840 (55%)	868 (57%)
Male	1,336 (44%)	682 (45%)	654 (43%)
Race			
White/Caucasian	1,759 (58%)	878 (58%)	881 (58%)
Asian	489 (16%)	245 (16%)	244 (16%)
Black/African American	360 (12%)	177 (12%)	183 (12%)
Other	223 (7%)	115 (8%)	108 (7%)
Hispanic/Latino	117 (4%)	61 (4%)	56 (4%)
More than one race	72 (2%)	36 (2%)	36 (2%)
Middle Eastern	24 (1%)	10 (1%)	14 (1%)
Cancer type			
GI	787 (26%)	385 (25%)	402 (26%)
Breast	776 (25%)	372 (24%)	404 (27%)
GU	421 (14%)	229 (15%)	192 (13%)
Heme	379 (12%)	197 (13%)	182 (12%)
Lung	194 (6%)	98 (6%)	96 (6%)
H&N	189 (6%)	98 (6%)	91 (6%)
Gyn	142 (5%)	70 (5%)	72 (5%)
Skin	102 (3%)	48 (3%)	54 (4%)
Other	42 (1%)	19 (1%)	23 (2%)
Brain	12 (0%)	6 (0%)	6 (0%)
Insurance			
Commercial	1,441 (47%)	741 (49%)	700 (46%)
Medicare	1,166 (38%)	574 (38%)	592 (39%)
Medicaid	208 (7%)	81 (5%)	127 (8%)
Charity	193 (6%)	90 (6%)	103 (7%)
Self-pay	36 (1%)	36 (2%)	0 (0%)

Abbreviations: GU, genitourinary; Gyn, gynecologic; Heme, hematologic; H&N, head and neck.

In cohort 1, the most common OP-35 diagnosis during an event was pain, representing 32% of diagnoses. Infectious causes, including sepsis, fever, pneumonia, and neutropenia, comprised 35% of all diagnoses. Nausea or emesis was diagnosed in 25% of events, and dehydration was diagnosed in 9% of events. Each patient could have multiple events, and each event could have multiple diagnoses (Table 2).

**Table 2:** ED diagnoses: summary of cohort 1

Diagnosis	Events, *n* (total events = 2,694)	Percentage of total diagnoses	Percentage of total events^a^
Pain	1,345	32%	50%
Fever	642	15%	24%
Sepsis	476	11%	18%
Emesis	366	9%	14%
Nausea	317	8%	12%
Anemia	261	6%	10%
Dehydration	239	6%	9%
Pneumonia	211	5%	8%
Diarrhea	167	4%	6%
Neutropenia	151	4%	6%
Total diagnoses	4,175		

a Each patient could have multiple events, and each event could have multiple diagnoses. Therefore, percentages will not sum to 100%.

**Figure 2:**
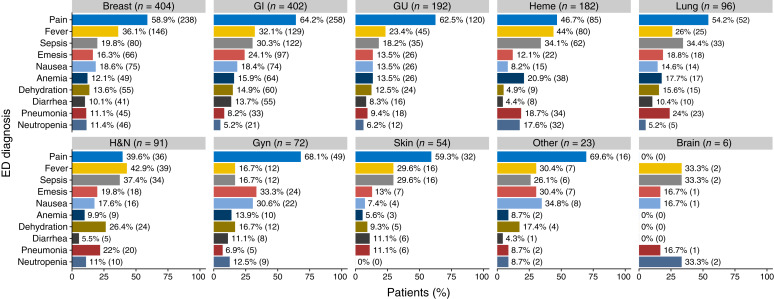
Percentage of patients in cohort 1 who experienced one or more events by ED diagnosis. Percentage (*n*) of patients who experienced ≥1 event are displayed for each cancer type. GU, genitourinary; Gyn, gynecologic; Heme, hematologic; H&N, head and neck.

Figure 2 summarizes the number of ED diagnoses by cancer type in cohort 1. Sixty-eight percent of patients with gynecologic cancer experienced one or more pain-related events compared with 40% patients with head and neck cancer.

Hispanics/Latinos accounted for only 4% of cohort 1 but had the highest average number of events (mean = 2.1; range = 1–10). Middle Eastern, more than one race, and Asians had the lowest mean number of events (1.3, 1.7, and 1.7, respectively).

**Figure 3:**
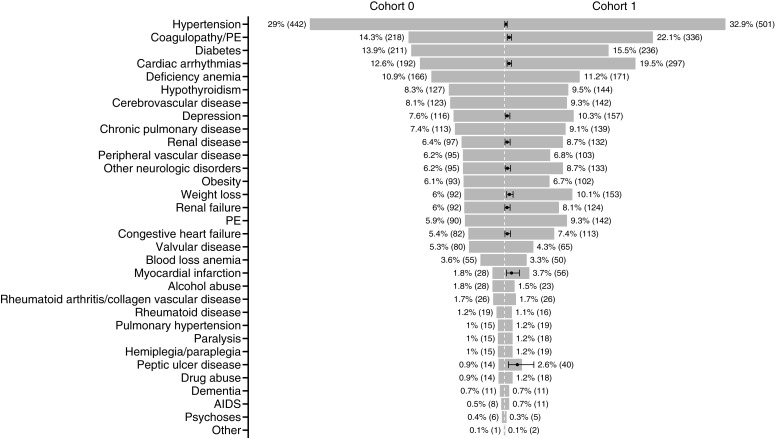
Comparison of patients with comorbidities between cohorts, percent (*n*). ORs with 95% CIs are displayed in black for statistically significant comorbidities. The dotted line corresponds to zero patients and an OR of 1. OR not in scale with number of patients. PE, pulmonary emboli.

In order to investigate whether medical comorbidities were associated with event risk, we matched this group 1:1 to a second cohort of ISCI patients by sex, age, race, and cancer type who did not have events. We started with 32 comorbidities as potential important covariates. Eleven comorbidities were selected based on statistically significant ORs to include in the initial model (Fig. 3). Conditional logistic regression with bidirectional stepwise variable selection was performed on the 11 comorbidities. Five were statistically significant (*P* < 0.05) and kept in the final model (coagulopathy/pulmonary emboli, cardiac arrhythmias, depression, weight loss, and myocardial infarction). Concordance equaled 0.58, which is equivalent to area under the ROC curve. The RF model had an accuracy of 56% with 95% CI 53%, 60%, and the SVM model was 55% accurate with 95% CI 52%, 59%. Sensitivity and specificity were 0.58 and 0.55, respectively, in the RF model. Sensitivity and specificity were 0.59 and 0.54, respectively, in the SVM model. Of the patients experiencing an event, 46% had at least one of the five comorbidities.

When we repeated the analysis with a subset of patients (*n* = 1,133 per cohort) who had one or more of the five potentially highly clinically preventable OP-35 conditions (pain, nausea, emesis, dehydration, or diarrhea), myocardial infarction was not significant anymore, but peptic ulcer disease was significant (Table 3).

**Table 3:** Conditional logistic regression models

Full study population (*n* = 1,522 per group)			Highly preventable OP-35 diagnoses^a^ (*n* = 1,133 per group)		
Comorbidity^b^	OR	*P*	Comorbidity^b^	OR	*P*
Coagulopathy and PE	1.5635	<0.001	Coagulopathy and PE	1.5408	<0.001
Cardiac arrhythmias	1.4935	<0.001	Cardiac arrhythmias	1.3706	0.009
Depression	1.3118	0.042	Depression	1.4017	0.026
Weight loss	1.4636	0.007	Weight loss	1.5221	0.011
Myocardial infarction	1.7837	0.021	Myocardial infarction	1.6767	0.069
Peptic ulcer disease	1.5581	0.067	Peptic ulcer disease	2.0422	0.032

Abbreviation: PE, pulmonary emboli.

a Regression was performed only on patients with one or more of the five highly preventable events (pain, emesis, nausea, dehydration, and diarrhea).

b Variables selected using bidirectional stepwise selection and Akaike information criterion.

## Discussion

The outpatient treatment of cancer with cytotoxic chemotherapy can cause significant acute toxicities in patients, posing a key clinical challenge to medical oncologists. When toxicities become severe, patients often require evaluation in the ED, significantly affecting the quality of life of patients with cancer and their families, incurring substantial medical costs, and presenting life-threatening complications. Our findings are consistent with those reported by Whitney and colleagues (14, 15), who observed high rates of hospitalization among patients with advanced cancers within the year following diagnosis, reinforcing the necessity of addressing these acute care needs.

Our findings revealed several important demographic and clinical risk factors for acute care visits among chemotherapy patients. Males were predicted to have 12% more visits than females. Insurance status also played a role, with patients on Medicaid or charity care exhibiting higher rates of acute care visits than those with commercial insurance or Medicare. Hispanic/Latino patients had the highest average number of events, whereas patients identifying as Middle Eastern, more than one race, or Asian had the lowest median number of events.

Comorbidity analysis highlighted five significant conditions associated with an increased risk of acute care visits: coagulopathy/pulmonary emboli, cardiac arrhythmias, depression, weight loss, and myocardial infarction. Approximately 46% of patients with an acute care event had at least one of these comorbidities. These findings emphasize the need for targeted interventions to manage these high-risk patients more effectively in the outpatient setting.

Our results align with previously reported data on hospitalization risk factors among those being treated for cancer. For instance, Whitney and colleagues identified significant rates (67%) of unplanned hospitalizations among patients with cancer, emphasizing the impact of comorbid conditions and the importance of early intervention. Additionally, the most frequent diagnoses seen for unplanned hospitalizations were GI cancers (hepatobiliary and pancreatic), similar to our findings (15). Daly and colleagues developed a risk model predicting potentially preventable acute care visits, identifying factors such as previous hospitalizations, stage of cancer, and specific comorbidities as critical predictors. Similar to our results, this study found that the most common symptoms that led to a potentially preventable acute care visit in the first 6 months after treatment initiation were pain, fever, and nausea/vomiting (16, 17).

Another study highlighted the importance of comorbidities and patient demographics in predicting chemotherapy-related hospitalizations and found some additional factors not analyzed in this study. This report found seven variables associated with hospitalizations, most notably age, Charlson comorbidity score, type of therapy received, and pretreatment lab results (18). Many of these were also previously identified, particularly the type of therapy and baseline lab values (19–21). Similarly, Delgado-Guay et al. (17) demonstrated that many ED visits (23%) among patients with advanced cancer receiving outpatient palliative care could be avoided with better symptom management and outpatient care strategies.

Our study identified significant demographic and comorbidity-related risk factors for requiring acute care following outpatient chemotherapy, corroborating and expanding on previous findings. For example, Csik and colleagues conducted a retrospective cohort analysis at a large urban academic medical center, identifying chronic obstructive pulmonary disease, congestive heart failure, renal failure, low neutrophil count, and low hemoglobin as correlates for ED visits within 90 days. Their risk model accounted for 46% of acute care visits, with a model C-statistic of 0.726 (22). Another study investigated readmissions for patients with acute leukemia, lymphoma, and multiple myeloma, finding that 21% of patients underwent unplanned readmissions within 30 days. They identified the absence of social work and behavioral health consultations as significant risk factors for acute care events (23).

As part of a “best practices” analysis, Handley and colleagues (3) proposed five key strategies for reducing unplanned inpatient care for patients with cancer by identifying patients at high risk for acute care interventions. These strategies include enhanced monitoring to identify these patients, care coordination, developing urgent cancer care tactics, standardized management, and early palliative care for high-risk patients. The authors recommended standardizing clinical pathways for symptom management to ensure that all clinical team members are treating chemotherapy toxicities in a similar and timely manner. Although these strategies may involve upfront costs, the researchers note that the long-term savings to the healthcare system may be realized years later.

Our study’s limitations include its retrospective design and lack of comprehensive social determinants of health data—factors known to affect the risk of acute care events (12). Goals-of-care information (adjuvant/neoadjuvant/palliative) was not easily accessible for our study. These data are often scattered across many sections of the electronic health records as free text, making it challenging to locate and extract for analysis. However, this could be an important factor to consider for future studies. Nonetheless, our findings highlight the need for targeted interventions to mitigate the risk of acute care visits among higher-risk patients identified in our analysis and provide additional information about comorbidities which can be considered in clinical decision-making in the management and treatment of patients with cancer.

During the data filtering process, less than 1% of patients were found to have only inpatient admissions during the study period (i.e., inpatient admissions but no ED visits). Therefore, we decided to focus our analysis only on ED visits. Further research should also incorporate a broader range of factors, including social determinants of health, to develop more comprehensive risk profiles and intervention strategies. Future interventions should focus on introducing risk-reducing monitoring in the outpatient setting for patients identified as higher risk.

This study underscores the critical need for improved outpatient management and monitoring strategies to address acute toxicities in chemotherapy patients. By leveraging the identified risk factors, healthcare providers could develop targeted interventions to mitigate the risk of acute care events, ultimately enhancing patient care and reducing the financial burden on the healthcare system.

## Supplementary Material

Supplementary Table 1 Frequency of events by authorizing provider specialty

Supplementary Table 2 Subset of study population showing only gastrointestinal and genitourinary cancer patients
